# Applications of Magnetosomes Synthesized by Magnetotactic Bacteria in Medicine

**DOI:** 10.3389/fbioe.2014.00005

**Published:** 2014-03-11

**Authors:** Edouard Alphandéry

**Affiliations:** ^1^Nanobacterie SARL, Paris, France^†^; ^2^Institut de Minéralogie et de Physique des Milieux Condensés, Université Pierre et Marie Curie, Paris, France

**Keywords:** magnetosomes, magnetic hyperthermia, magnetotactic bacteria, cancer, heat therapy, thermotherapy

## Abstract

Magnetotactic bacteria belong to a group of bacteria that synthesize iron oxide nanoparticles covered by biological material that are called magnetosomes. These bacteria use the magnetosomes as a compass to navigate in the direction of the earth’s magnetic field. This compass helps the bacteria to find the optimum conditions for their growth and survival. Here, we review several medical applications of magnetosomes, such as those in magnetic resonance imaging (MRI), magnetic hyperthermia, and drug delivery. Different methods that can be used to prepare the magnetosomes for these applications are described. The toxicity and biodistribution results that have been published are summarized. They show that the magnetosomes can safely be used provided that they are prepared in specific conditions. The advantageous properties of the magnetosomes compared with those of chemically synthesized nanoparticles of similar composition are also highlighted.

## Introduction

Magnetosomes are intracellular structures produced by magnetotactic bacteria, which comprise magnetic nanoparticles surrounded by lipid bilayer membrane. They have attracted much attention for biotechnological applications. This is due to a series of appealing properties summarized below that are not usually found in chemically synthesized nanoparticles.

(i) The magnetosomes are magnetic nanoparticles, which possess a narrow size distribution and uniform morphology when the magnetotactic bacteria are cultivated in optimum conditions, i.e., essentially using a low oxygen concentration (varied between 0.25 and 10 mbar, Heyen and Schüler, 2003) during the growth. In these conditions, the magnetosome size distribution can be as small as ~10 nm with magnetosome sizes typically lying between 45 and 55 nm for the most commonly studied species of magnetotactic bacteria (AMB-1 and MSR-1) (Bazylinski and Frankel, 2004; Taylor and Barry, 2004).
(ii) The core of the magnetosomes is usually composed of magnetite (Fe_3_O_4_) that can oxidize into maghemite (γFe_2_O_3_). The magnetosome core is also usually of high levels of purity and crystallinity (Bazylinski and Frankel, 2004).
(iii) The magnetosomes are usually large single magnetic domain nanoparticles. This leads to a magnetic moment that is thermally stable at physiological temperature. Therefore, it produces better magnetic properties than those found in chemically synthesized iron oxide nanoparticles that are usually superparamagnetic and possess a thermally unstable magnetic moment. It also yields high values of the coercivity (*H*_c_ ~ 20–40 mT) and ratio between the remanent and saturation magnetization (*M*_r_/*M*_s_ ~ 0.4–0.5) (Pan et al., 2005; Alphandéry et al., 2008). In specific conditions described below, these magnetic properties result in higher heating capacities and better magnetic resonance imaging (MRI) contrast agents for the magnetosomes than for chemically synthesized nanoparticles.
(iv) The magnetosomes are usually arranged in chains inside the bacteria. This arrangement is stable enough to be preserved even after disrupting the bacteria to isolate the magnetosomes. Such arrangement is appealing since it prevents aggregation and yields a high rate of internalization within human cells, two properties that are usually desired for medical applications (Alphandéry et al., 2011b, 2012a).
(v) The magnetosomes are covered by biological material made of a majority of lipids and a minority of proteins. This biological coating results in negatively charged magnetosomes with a good dispersion in water (Alphandéry et al., 2011b, 2012a). By contrast, chemically synthesized nanoparticles are not naturally coated and need to be stabilized, for example, by being covered with dextran or PEG molecules. This usually makes their synthesis more complicated than that of the magnetosomes.
(vi) The magnetosomes can easily be functionalized, due to the presence of various chemical groups at their surface (Sun et al., 2011).
(vii) Methods have been published that enable to produce a large quantity of magnetosomes up to 170 mg/L/day of magnetosomes (Matsunaga et al., 1990, 1996; Yang et al., 2001; Heyen and Schüler, 2003; Sun et al., 2008a; Liu et al., 2010; Zhang et al., 2011; Alphandéry et al., 2012b).
(viii) When they are prepared in specific conditions, the magnetosomes possess a high biocompatibility and a low toxicity (Xiang et al., 2007; Sun et al., 2010).
(ix) Finally, magnetosomes are obtained by cultivating magnetotactic bacteria in a growth medium, which is not toxic (for example, ATCC medium 1653 for the AMB-1 species). This contrasts with the use of toxic products often used during the preparation of chemically synthesized nanoparticles.

## Preparation of Suspensions of Magnetotactic Bacteria and Bacterial Magnetosomes

**Figure 1:**
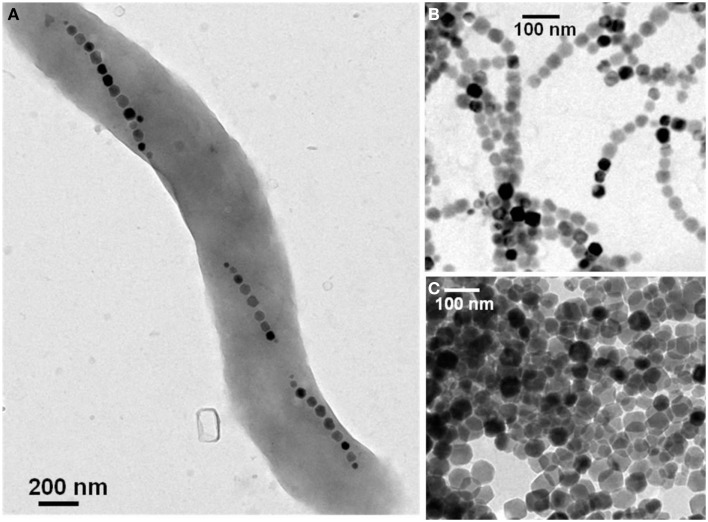
**Transmission electron microscopy images of a single magnetotactic bacterium (A) of chains of magnetosomes extracted from whole magnetotactic bacteria (B) of individual magnetosomes detached from the chains by heat and SDS treatment (C)**.

Depending on the type of application, different types of suspensions containing either magnetotactic bacteria or isolated magnetosomes can be prepared. For example, for targeting tumors, it has been suggested to use suspensions of living magnetotactic bacteria, which are administered intravenously and are naturally attracted by the anoxic environment of the tumor (Benoit et al., 2009). However, the use of living magnetotactic bacteria for medical applications will unlikely be accepted by regulatory agencies (FDA in the USA, EMA in Europe) and this review focuses on magnetosomes that will more likely be accepted for clinical trials. For treating cancers using magnetic hyperthermia, it has been suggested to use suspensions containing chains of magnetosomes (chains of magnetic nanoparticles) extracted from magnetotactic bacteria (Alphandéry et al., 2011a,b, 2012a, 2013). For other applications, the magnetosomes that have been used have been isolated from magnetotactic bacteria and treated to remove biological material surrounding them. They have then been coated with lipids for stabilization (Yoshino et al., 2007). To prepare suspensions containing living whole magnetotactic bacteria, AMB-1, MSR-1, or MS-1 strains can be purchased from the ATCC or DSMZ culture collection with a growth protocol, which is provided. A TEM (transmission electron microscopic) image of a typical whole magnetotactic bacterium containing several chains of magnetosomes is presented in Figure 1A. Among the different strains of magnetotactic bacteria, MSR-1 has achieved the highest yield of magnetosome production (170 mg/L/day) (Zhang et al., 2011), and therefore seems to be a promising strain for medical applications, which require a large quantity of magnetosomes. To obtain suspensions containing extracted chains of magnetosomes such as those shown in Figure 1B, the latter can be isolated from magnetotactic bacteria using either sonication (Taoka et al., 2006; Sun et al., 2008b; Alphandéry et al., 2011b), a treatment with sodium hydroxide (Philipse and Maas, 2002), with a French press (Grünberg et al., 2004; Matsunaga et al., 2007; Xiang et al., 2007), or with a pressure homogenizer (Guo et al., 2011; Tang et al., 2012). Different methods have also been suggested to purify the suspension of magnetosomes after extraction involving either magnetic separation of the magnetosomes from the cellular debris (Grünberg et al., 2001; Alphandéry et al., 2011b, 2012a), a treatment with proteinase K to remove surface proteins (Guo et al., 2011), phenylmethylsulfonyl fluoride to inhibit the activity of the protease, DNase I to remove DNA (Sun et al., 2007). The most commonly used method of sterilization for magnetosome suspensions is gamma rays (Guo et al., 2011). The magnetosomes can be stabilized in water. Before being administered to human, the suspensions of magnetosomes also need to be characterized. The biological material surrounding the magnetosomes can be characterized using chromatography, infrared spectroscopy, SDS Page, and mass spectroscopy (Grünberg et al., 2004). TEM can be used to measure the size of the magnetosomes and to verify the high level of crystallinity of the magnetosome core. Magnetic measurements could also be carried out to detect the Verwey transition, which would reveal the presence of magnetite in the magnetosomes (Alphandéry et al., 2008). Finally, it is also possible to obtain suspensions of individual magnetosomes isolated from magnetotactic bacteria (Figure 1C), in which most of the biological material has been removed by heating the suspensions of magnetosomes during 5 h at 90°C in the presence of 1% SDS (Alphandéry et al., 2011b, 2012a).

## Toxicity and Biodistribution of Bacterial Magnetosomes

The cytotoxicity of bacterial magnetosomes was found to depend strongly on the type of cell tested. For Hepatoma H22 cells (liver cancer cells) and promyelocytic HL60 cells (leukemia cells), the bacterial magnetosomes began to show signs of cytotoxicity at relatively low magnetosome concentrations of 9 μg/mL (Sun et al., 2010). On the other hand, for fibroblasts and breast cancer cells, such as EMT-6 cells or MDA-MB-231 cells, the magnetosomes were not cytotoxicity below ~1 mg/mL (Xiang et al., 2007; Sun et al., 2010; Alphandéry et al., 2011b). The cytotoxicity was also found to depend on the incubation time (Alphandéry et al., 2008). Indeed, for magnetosome concentrations higher than 1 mg/mL, cytotoxicity was found to be low when the incubation time was shorter than 24 h. On the other hand, for magnetosome concentrations lower than 1 mg/mL, cytotoxicity remained low even for longer incubation times than 24 h. The cytotoxicity of the magnetosomes seems to be comparable to that of superparamagnetic iron oxide nanoparticles (SPION), which were found to be cytotoxic above a concentration of 100 μg/mL (Karlsson et al., 2008; Ankamwar et al., 2010; Singh et al., 2010). The acute toxicity of the bacterial magnetosomes was also studied. It was found that for an intravenous administration of bacterial magnetosomes, rats could survive for a maximum quantity of magnetosomes administered of 480 mg/kg (Liu et al., 2012). This quantity of nanoparticles is higher than that of 135 mg/kg found for SPION (Liu et al., 2012). The immunotoxicity of the magnetosomes was also reported. For that, 1 mg of a suspension of bacterial magnetosomes (magnetic nanoparticles) was administered in the ears of rabbits and rabbit temperatures were monitored (Sun et al., 2010). It was found that rabbit temperatures did not increase following the administration of the suspensions of magnetosomes. This result together with other findings presented in reference (Sun et al., 2010) support the idea that the magnetosomes are not pyrogenic when they are prepared in the specific conditions described in Sun et al. (2010).

Regarding the biodistribution of the magnetosomes, different situations can arise. The magnetosomes could be metabolized by the organism and transformed into free iron. They may also remain in the form of crystallized nanoparticles. In both cases, free iron or crystallized magnetosomes can either accumulate in the organism or be eliminated in the feces or urines. In order to understand the biodistribution of the magnetosomes, the latter have been mixed with proteins from bovine pancreas that simulate the activity of lysosomes. It was observed that the magnetosomes were degraded by the proteases after 28 days, which suggests that lysosomes degrade the magnetosomes (Liu et al., 2012). Magnetosomes have also been administered intravenously in rats (Sun et al., 2009) and mice (Liu et al., 2012), and it was found that they end up in the lysosomes of liver and spleen (Sun et al., 2009; Liu et al., 2012). These results suggest that the reticuloendothelial system removes the magnetosomes from bloodstream and that the magnetosomes are then transformed into free iron (Sun et al., 2009). In this study (Sun et al., 2009), the magnetosomes were neither found in the feces nor in the urine or rats. However, in other studies, magnetosomes have been found in the feces of rats following intratumoral administration (Alphandéry et al., 2011b), or intravenous administration, as observed by TEM observation of the feces (unpublished results obtained by us). We can conclude that after being administered in the organism, a portion of the magnetosomes is most probably transformed into free iron while another portion remains in a crystallized form and is eliminated in the feces. However, more studies are necessary to better understand the magnetosome biodistribution.

## Applications of Magnetosomes in MRI, Magnetic Hyperthermia, and Drug Delivery

### Magnetic resonance imaging

Several studies report the use of bacterial magnetosomes as positive or negative contrast agents. Benoit et al. (2009) have shown using MRI that magnetotactic bacteria have a natural tendency to target tumors in mice when they are administered intravenously. In this study, magnetotactic bacteria were visualized in tumors using MRI. A portion of the magnetosomes was shown to generate *T*_1_ (longitudinal relaxation times)-weighted positive contrast, improving the visualization of the magnetotactic bacteria in the tumors. Another interesting aspect of this report resides in the finding that small magnetosomes of ~25 nm in mean sizes produce a positive contrast while large magnetosomes of mean sizes ~50 nm do not produce such contrast. For the small magnetosomes, the *T*_1_-weighted MRI signal is also found to increase with increasing bacterial concentrations provided the bacterial concentration remains below a threshold of 0.5 10^10^ cells/mL. Above 0.5×10^10^ cells/mL, the *T*_1_-weighted MRI signal decreases due to the competing *T*_2_ (transverse relaxation times) effect. In general, good contrast agents are characterized by very high relaxivities (the inverse of the *T*_2_ relaxation time, usually designated as *r*_2_) and very short values of *T*_2_. Such high values of *r*_2_ can be reached with the magnetosomes. Indeed, it has been shown that both magnetosomes enclosed in a gel and ferrimagnetic nanoparticles different from ferridex and with similar properties than the magnetosomes possess values of *r*_2_ as high as 1175 and 324 mM s^−1^, respectively (Hu et al., 2010; Lee et al., 2011). These two values are larger than the value of *r*_2_ ~ 133 mM s^−1^ found for chemically synthesized nanoparticles ferridex, which are currently approved and tested in the clinic as contrast agents for MRI application (Lee et al., 2011).

### Magnetic hyperthermia

Magnetosomes are also good candidates to treat cancers using magnetic hyperthermia. Magnetic hyperthermia is a technique in which magnetic nanoparticles are administered (or sent) to tumors and then heated under the application of an alternating magnetic field. The heat induces anti-tumor activity. In order to be efficient for magnetic hyperthermia, the nanoparticles therefore need to produce a large amount of heat. The magnetosomes possess good heating properties essentially due to their large sizes, their ferromagnetic behavior at physiological temperature and their high level of crystallinity. For ferrimagnetic nanoparticles, the quantity of heat generated under the application of an alternating magnetic field is essentially proportional to the area of their hysteresis loop, which increases with increasing nanoparticle sizes. Indeed, in most cases, *H*_c_ and *M*_r_/*M*_s_ that are proportional to the area of the hysteresis loops, increase with increasing nanoparticle sizes. The amount of heat produced by the magnetosomes has been estimated by measuring magnetosome losses per cycles, which are defined as the magnetosome SAR (specific absorption rates) divided by the frequency of the applied magnetic field. The magnetosome losses per cycle were found to increase with increasing magnetic field strength from 0.1 to 0.2 J/kg (joules per kilogram of iron contained in the heated magnetosomes) for a magnetic field strength of 6 mT (Hergt et al., 2005, 2008; Dutz et al., 2007; Timko et al., 2009, 2013), up to 0.5–1 J/kg for a magnetic field strength of 12 mT (Hergt et al., 2005, 2008; Dutz et al., 2007; Sun et al., 2009). These values are larger than those of SPION essentially when the magnetic field strength is higher than 10 mT (Alphandéry et al., 2011a).

The heating mechanisms of the magnetosomes have also been studied (Alphandéry et al., 2011a). The aim of this study (Arakaki et al., 2008), was to determine which type of magnetosomes between the magnetosomes contained in whole magnetotactic bacteria, the chains of magnetosomes isolated from magnetotactic bacteria, and the individual magnetosomes detached from the chains by heat and SDS treatments, is the most suitable candidate for the magnetic hyperthermia treatment of tumors. There are essentially two mechanisms that can produce heat when magnetosomes are exposed to an alternating magnetic field. They are either due to the reversal of the magnetosome magnetic moment or to the physical rotation of the magnetosomes under the application of an alternating magnetic field. In order to eliminate the contribution of the rotation to the heating mechanism of the magnetosomes, suspensions of whole magnetotactic bacteria that do not produce heat by rotation have been exposed to an alternating magnetic field (Alphandéry et al., 2011a). For such suspensions of whole magnetotactic bacteria, losses per cycles of 1.1 J/kg_Fe_ at 23 mT and of 8 J/kg_Fe_ at 88 mT were measured. In order to enable the rotation of the chains of magnetosomes, the latter were extracted from magnetotactic bacteria by sonication. For these extracted chains of magnetosomes, the losses per cycle increased to 5 J/kg_Fe_ at 23 mT and to 11 J/kg_Fe_ at 83 mT (Alphandéry et al., 2011a), showing that the rotation of the chains of magnetosomes contributes to the heating mechanism. Finally, the magnetosomes have been detached from the chains by heat and SDS treatment to produce suspensions of individual magnetosomes. The values of the SAR obtained for the individual magnetosomes (5 J/kg_Fe_ at 23 mT and 9 J/kg_Fe_ at 83 mT (Alphandéry et al., 2011a)) were relatively similar to those of the chains of magnetosomes. Therefore, it was not possible to decide which one between the chains of magnetosomes or the individual magnetosomes would be the best candidate for *in vivo* magnetic hyperthermia treatments of tumors (Alphandéry et al., 2011a). Therefore both types of magnetosomes were tested *in vivo*.

**Figure 2:**
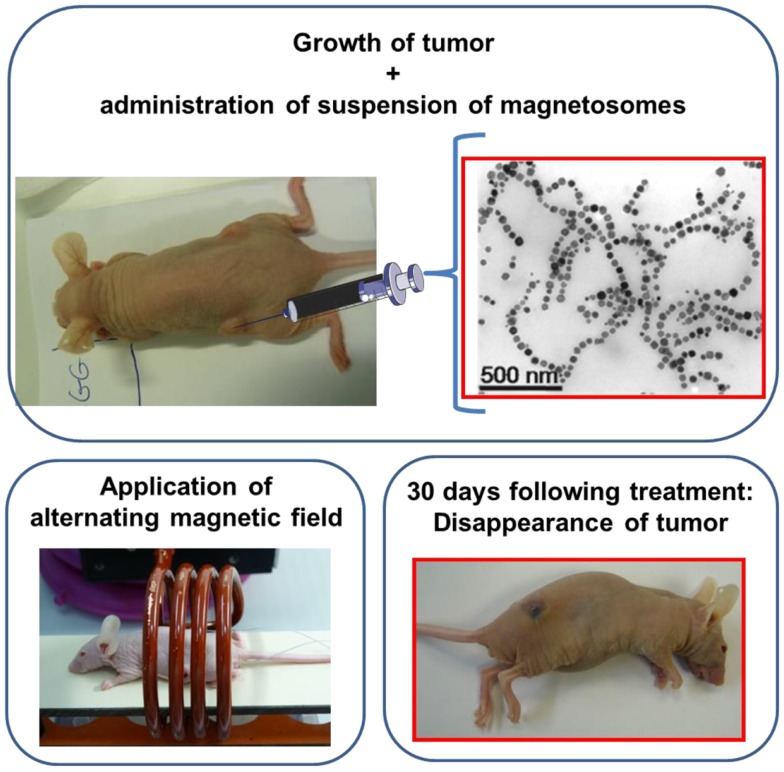
**Schematic diagram showing the treatment of a mouse using magnetic hyperthermia**. The mouse has a breast tumor xeno-grafted under its skin. A suspension of magnetosomes is administered at the center of the tumor; the mouse is then positioned inside a coil where an alternating magnetic field is applied three times during 20 min. The tumor disappears 30 days following the treatment as can be seen in the bottom right photograph.

In order to evaluate the anti-tumor activity of the magnetosomes (Alphandéry et al., 2011b), 100 μL of suspensions containing either individual magnetosomes or chains of magnetosomes at a concentration of 10 mg/mL were administered at the center of MDA-MB-231 breast tumors xeno-grafted under the skin of mice following the protocol illustrated in the schematic diagram of Figure 2. The mice were then exposed to an alternating magnetic field of average field strength ~20 mT and frequency 198 kHz three times during 20 min. This produced an increase in the tumor temperature up to ~43°C. The treatment with the chains of magnetosomes yielded the total disappearance of the tumor 30 days following the treatment in several mice (Figure 2), while that using the individual magnetosomes did not produce significant anti-tumor activity (Alphandéry et al., 2011b). The efficacy of the treatment was attributed on the one hand to the internalization of the chains of magnetosomes inside the tumor cells that enabled intracellular heating and hence efficient tumor cell destruction. On the other hand, the efficacy of the chains of magnetosomes was reported to arise from their homogenous distribution throughout the tumor, which is mostly due to their low level of aggregation.

### Drug delivery

Due to the presence of various chemical groups at the surface of the magnetosomes, it is possible to conjugate drugs such as doxorubicin to the magnetosome surface (Sun et al., 2007, 2008b). Magnetosomes to which doxorubicin is conjugated have been tested as anti-tumor agents against hepatic cancer. It has been shown that by conjugating doxorubicin to the magnetosomes, it was possible to slightly increase the anti-tumor activity from 79% for doxorubicin alone up to 87% for doxorubicin bound to the magnetosomes (Sun et al., 2007, 2008b). The advantage of using the magnetosomes is mainly due to the decrease in toxicity. While doxorubicin is highly toxic when it is used alone with a mortality rate of 80%, doxorubicin bound to the magnetosomes is much less toxic with a mortality rate of 20% (Sun et al., 2007, 2008b). Therefore, there is a large increase in the benefit to risk ratio when doxorubicin is conjugated to the magnetosomes, showing the potential of drugs conjugated to magnetosomes for cancer treatments.

## Other Medical Applications of Bacterial Magnetosomes

Magnetosomes can be used for other applications, for example, to detect nucleotide polymorphism, which is useful to diagnose diseases such as cancer, hypertension, or diabetes, to separate cells or to detect DNA (Arakaki et al., 2008). To separate cells, magnetic beads or SPION have been tested. However, these two types of magnetic materials present drawbacks. Magnetic beads are large and hence prevent cells from dividing and proliferating correctly. SPION on the other hand are only weakly magnetic due to their unstable magnetic moment at physiological and room temperatures, which makes them poorly efficient to separate cells. By contrast, the magnetosomes are of smaller sizes than the magnetic beads and are more strongly magnetic than the SPION due to their ferrimagnetic properties. This makes them ideal candidates for applications in cell separation (Arakaki et al., 2008). The magnetosomes have also been used for immunoassays, for example, to detect small molecules such as pollutant, hormones, or toxic detergents. These molecules have been attached to the magnetosome surface using antibodies that specifically bind to them. The complex formed by the magnetosomes and these molecules has then been detected (Arakaki et al., 2008). Finally, magnetosomes have been used to extract DNA. For that, they have been modified and covered with layers of aminosilanes that link DNA. The complex formed by the magnetosomes and DNA has been bound to a magnetic column and DNA has been collected by elution with a phosphate buffer (Arakaki et al., 2008).

In conclusion, we have presented in this review several medical applications of the magnetosomes and we have also briefly described a few methods that can be used to prepare the magnetosomes for these applications.

## Conflict of Interest Statement

The author declares that the research was conducted in the absence of any commercial or financial relationships that could be construed as a potential conflict of interest.

## References

[B1] Alphandéry E. Chebbi I. Guyot F. Durand-Dubief M. (2013). Use of bacterial magnetosomes in the magnetic hyperthermia treatment of tumours: a review. Int. J. Hyperthermia 29, 801–809 10.3109/02656736.2013.821527 24024595

[B2] Alphandéry E. Faure S. Raison L. Duguet E. Howse P. A. Bazylinski D. A. (2011a). Heat production by bacterial magnetosomes exposed to an oscillating magnetic field. J. Phys. Chem. C 115, 18–22 10.1021/jp104580t

[B3] Alphandéry E. Faure S. Seksek O. Guyot F. Chebbi I. (2011b). Chains of magnetosomes extracted from AMB-1 magnetotactic bacteria for application in alternative magnetic field cancer therapy. ACS Nano 5, 6279–6296 10.1021/nn201290k 21732678

[B4] Alphandéry E. Guyot F. Chebbi I. (2012a). Preparation of chains of magnetosomes, isolated from *Magnetospirillum magneticum* strain AMB-1 magnetotactic bacteria, yielding efficient treatment of tumors using magnetic hyperthermia. Int. J. Pharm. 434, 444–452 10.1016/j.ijpharm.2012.06.015 22698862

[B5] Alphandéry E. Amor M. Guyot F. Chebbi I. (2012b). The effects of iron-chelating agents on *Magnetospirillum magneticum* strain AMB-1: stimulated growth and magnetosome production and improved magnetosome heating properties. Appl. Micriobiol. Biotechnol. 96, 663–670 10.1007/s00253-012-4199-5 22707052

[B6] Alphandéry E. Ngo A. T. Lefèvre C. Lisiecki I. Wu L. F. Pileni M. P. (2008). Difference between the magnetic properties of the magnetotactic bacteria and those of the extracted chains of magnetosomes: influence of the distance between the chains of magnetosomes. J. Phys. Chem. C 112, 12304–12309 10.1021/jp800408t

[B7] Ankamwar B. Lai T. C. Huang J. H. Liu R. S. Hsiao M. Chen C. H. (2010). Biocompatibility of Fe(3)O(4) nanoparticles evaluated by in vitro cytotoxicity assays using normal glia and breast cancer cells. Nanotechnology 21, 75102 10.1088/0957-4484/21/7/075102 20090199

[B8] Arakaki A. Nakazawa H. Nemoto M. Mori T. Matsunaga T. (2008). Formation of magnetite by bacteria and its application. J. R. Soc. Interface 5, 977–999 10.1098/rsif.2008.0170 18559314 PMC2475554

[B9] Bazylinski D. A. Frankel R. (2004). Magnetosome formation in prokaryotes. Nat. Rev. Microbiol. 2, 217–230 10.1038/nrmicro842 15083157

[B10] Benoit M. Mayer D. Barak Y. Chen I. Y. Hu W. Cheng Z. (2009). Visualizing implanted tumors in mice with magnetic resonance imaging using magnetotactic bacteria. Clin. Cancer Res. 15, 5170–5177 10.1158/1078-0432.CCR-08-3206 19671860 PMC3409839

[B11] Dutz S. Hergt R. Mürbe J. Müller R. Zeisberger M. Andrä W. (2007). Hysteresis losses of magnetic nanoparticle powders in the single domain size range. J. Magn. Magn. Mater. 308, 305–312 10.1088/0953-8984/20/38/385214 21693832

[B12] Grünberg K. Müller E.-C. Otto A. Reska R. Linder D. Kube M. (2004). Biochemical and proteomic analysis of the magnetosome membrane in *Magnetospirillum gryphiswaldense*. Appl. Environ. Microbiol. 70, 1040–1050 10.1128/AEM.70.2.1040-1050.2004 14766587 PMC348919

[B13] Grünberg K. Wawer C. Tebo B. M. Schüler D. (2001). A large gene cluster encoding several magnetosome proteins is conserved in different species of magnetotactic bacteria. Appl. Environ. Microbiol. 67, 4573–4582 10.1128/AEM.67.10.4573-4582.2001 11571158 PMC93205

[B14] Guo F. Liu Y. Chen Y. Tang T. Jiang W. Li Y. (2011). A novel rapid and continuous procedure for large-scale purification of magnetosomes from *Magnetospirillum gryphiswaldense*. Appl. Microbiol. Biotechnol. 90, 1277–1283 10.1007/s00253-011-3189-3 21360144

[B15] Hergt R. Dutz S. Roder M. (2008). Effects of size distribution on hysteresis losses of magnetic nanoparticles for hyperthermia. J Phys Condens Matter 20, 385214 10.1088/0953-8984/20/38/385214 21693832

[B16] Hergt R. Hiergeist R. Zeisberger M. Schüler D. Heyen U. Hilger I. (2005). Magnetic properties of bacterial magnetosomes as potential diagnostic and therapeutic tools. J. Magn. Magn. Mater. 293, 80–86 10.1016/j.jmmm.2005.01.047

[B17] Heyen U. Schüler D. (2003). Growth and magnetosome formation by microaerophilic *Magnetospirillum* strains in oxygen-controlled fermentor. Appl. Microbiol. Biotechnol. 61, 536–544 10.1007/s00253-002-1219-x 12764570

[B18] Hu L. L. Zhang F. Wang Z. You X. F. Nie L. Wang H. X. (2010). Comparison of the 1H NMR relaxation enhancement produced by bacterial magnetosomes and synthetic iron oxide nanoparticles for potential use as MR molecular probes. IEEE Trans. Appl. Supercond. 20, 822–825 10.1109/TASC.2010.2041218

[B19] Karlsson H. L. Cronholm P. Gustafsson J. Moller L. (2008). Copper oxide nanoparticles are highly toxic: a comparison between metal oxide nanoparticles and carbon nanotubes. Chem. Res. Toxicol. 21, 1726–1732 10.1021/tx800064j 18710264

[B20] Lee N. Kim H. Choi S. H. Park M. Kim D. Kim H.-C. (2011). Magnetosome-like ferromagnetic iron oxide nanocubes for highly sensitive MRI of single cells and transplanted pancreatic islets. Proc. Natl. Acad. Sci. U.S.A. 108, 2662–2667 10.1073/pnas.1016409108 21282616 PMC3041081

[B21] Liu R.-T. Liu J. Tong J.-Q. Tang T. Kong W.-C. Wang X.-W. (2012). Heating effect and biocompatibility of bacterial magnetosomes as potential materials used in magnetic fluid hyperthermia. Prog. Nat. Sci. Mater. Int. 22, 31–39 10.1016/j.pnsc.2011.12.006

[B22] Liu Y. Li G. R. Jiang W. Li Y. Li L. J. (2010). Large-scale production of magnetosomes by chemostat culture of *Magnetospirillum gryphiswaldense* at high cell density. Microb. Cell Fact. 9, 99 10.1186/1475-2859-9-99 21144001 PMC3019156

[B23] Matsunaga T. Maeda Y. Yoshino T. Takeyama H. Takahashi M. Ginya H. (2007). Fully automated immunoassay for detection of prostate-specific antigen nano-magnetic beads and micro-polystyrene bead composites, ‘Beads on Beads’. Anal. Chim. Acta 597, 331–339 10.1016/j.aca.2007.05.065 17683747

[B24] Matsunaga T. Tadokora F. Nakamura N. (1990). Mass culture of magnetic bacteria and their application to flow type immunoassays. IEEE Trans. Magn. 26, 1557–1559 10.1109/20.104444

[B25] Matsunaga T. Tsujimura N. Kamiya S. (1996). Enhancement of magnetic particle production by nitrate and succinate fed-batched culture of *Magnetospirillum* sp. AMB-1. Biotechnol. Tech. 10, 495–500 10.1007/BF00159513

[B26] Pan Y. Peterson N. Winklhofer M. Davila A. F. Liu Q. Frederichs T. (2005). Rock magnetic properties of uncultured magnetotactic bacteria. Earth Planet. Sci. Lett. 237, 311–325 10.1016/j.epsl.2005.06.029

[B27] Philipse A. P. Maas D. (2002). Magnetic colloids from magnetotactic bacteria: chain formation and colloidal stability. Langmuir 18, 9977–9984 10.1021/la0205811

[B28] Singh N. Jenkins G. J. S. Asadi R. Doak S. H. (2010). Potential toxicity of superparamagnetic iron oxide nanoparticles (SPION). Nano Rev. 1, 5358–5372 10.3402/nano.v1i0.5358 22110864 PMC3215220

[B29] Sun J. Li Y. Liang X.-J. Wang P. C. (2011). Bacterial magnetosome: a novel biogenetic magnetic targeted drug carrier with potential multifunctions. J. Nanomater. 2011:469031, 13 p. 10.1155/2011/469031 22448162 PMC3310401

[B30] Sun J. Tang T. Duan J. Xu P.-X. Wang Z. Zhang Y. (2010). Biocompatibility of bacterial magnetosomes: acute toxicity, immunotoxicity and cytotoxicity. Nanotoxicology 4, 271–283 10.3109/17435391003690531 20795909

[B31] Sun J.-B. Duan J.-H. Dai S.-L. Ren J. Zhang Y.-D. Tian J.-S. (2007). In vitro and in vivo antitumor effects of doxorubicin loaded with bacterial magnetosomes (DBMs) on H22 cells: the magnetic bio-nanoparticles as drug carriers. Cancer Lett. 258, 109–117 10.1016/j.canlet.2007.08.018 17920762

[B32] Sun J.-B. Wang Z.-L. Duan J.-H. Ren J. Yang X.-D. Dai S.-L. (2009). Targeted distribution of bacterial magnetosomes isolated from *Magnetospirillum gryphiswaldense* MSR-1 in healthy Sprague-Dawley rats. J. Nanosci. Nanotechnol. 9, 1881–1885 10.1166/jnn.2009.410 19435053

[B33] Sun J.-B. Zhao F. Tang T. Jiang W. Tian J.-S. Li J.-L. (2008a). High-yield growth and magnetosome formation by *Magnetospirillum gryphiswaldense* MSR-1 in an oxygen-controlled fermentor supplied solely with air. Appl. Microbiol. Biotechnol. 79, 389–397 10.1007/s00253-008-1453-y 18425510

[B34] Sun J.-B. Duan J.-H. Dai S.-L. Ren J. Guo L. Jiang W. (2008b). Preparation and anti-tumor efficiency evaluation of doxorubicin-loaded bacterial magnetosomes: magnetic nanoparticles as drug carriers isolated from *Magnetospirillum gryphiswaldense*. Biotechnol. Bioeng. 101, 1313–1320 10.1002/bit.22011 18980188

[B35] Tang T. Zhang L. Gao R. Dai Y. Meng F. Li Y. (2012). Fluorescence imaging and targeted distribution of bacterial magnetic particles in nude mice. Appl. Microbiol. Biotechnol. 94, 495–503 10.1007/s00253-012-3981-8 22395909

[B36] Taoka A. Asada R. Sasaki H. Anzawa K. Wu L.-F. Fukumori Y. (2006). Spatial localizations of Mam22 and Mam12 in the magnetosomes of *Magnetospirillum magnetotacticum*. J. Bacteriol. 188, 3805–3812 10.1128/JB.00020-06 16707673 PMC1482926

[B37] Taylor A. P. Barry J. C. (2004). Magnetosomal matrix: ultrafine structure may template biomineralization of magnetosomes. J. Microscopy 213, 180–197 10.1111/j.1365-2818.2004.01287.x 14731301

[B38] Timko M. Dzarova A. Skumiel A. Jozefcak A. Hornowski T. Gojzewski H. (2009). Magnetic properties and heating effect in bacterial magnetic nanoparticles. J. Magn. Magn. Mater. 321, 1521–1524 10.1016/j.jmmm.2009.02.077

[B39] Timko M. Molcan M. Hashim A. Skumiel A. Muller M. Gojzewski H. (2013). Hyperthermic effect in suspension of magnetosomes prepared by various methods. IEEE Trans. Magn. 49, 250–254 10.1109/TMAG.2012.2224098

[B40] Xiang L. Wei J. Jianbo S. Gulli W. Feng G. Ying L. (2007). Purified and sterilized magnetosomes from *Magnetospirillum gryphiswaldense* MSR-1 were not toxic to mouse fibroblasts in vitro. Lett. Appl. Microbiol. 45, 75–81 10.1111/j.1472-765X.2007.02143.x 17594464

[B41] Yang C.-D. Takeyama H. Tanaka T. Matsunaga T. (2001). Effects of growth medium composition, iron sources and atmospheric oxygen concentrations on production of luciferase-bacterial magnetic particle complex by a recombinant *Magnetospirillum magneticum* AMB-1. Enzyme Microb. Technol. 29, 13–19 10.1016/S0141-0229(01)00343-X 11427230

[B42] Yoshino T. Hirabe H. Takahashi M. Kuhara M. Takeyama H. Matsunaga T. (2007). Magnetic cell separation using nano-sized bacterial magnetic particles with reconstructed magnetosome membrane. Biotechnol. Bioeng. 101, 470–477 10.1002/bit.21912 18421798

[B43] Zhang Y. Zhang X. Jiang W. Li Y. Li J. (2011). Semicontinuous culture of *Magnetospirillum gryphiswaldense* MSR-1 cells in an autofermentor by nutrient-balanced and isosmotic feeding strategies. Appl. Environ. Microbiol. 77, 5851–5856 10.1128/AEM.05962-11 21724877 PMC3165407

